# Supplementary material for: Extended depth-resolved imaging through a thin scattering medium with PSF manipulation
Source: Sci Rep. 2018 Mar 15;8:4585. doi: 10.1038/s41598-018-22966-7 (PMC5854624; doi:10.1038/s41598-018-22966-7)
Supplement: Supplementary file 2 — Supplementary information [file 41598_2018_22966_MOESM2_ESM.doc]

SUPPLEMENTARY INFORMATION

**Extended depth-resolved imaging through a thin scattering medium with PSF manipulation**

Xiangsheng Xie^1, 2^, Huichang Zhuang^2^, Hexiang He^3, *^, Xiaoqing Xu^2^, Haowen Liang^2^, Yikun Liu^2^ and Jianying Zhou^2, *^

^1^Department of Physics, College of Science, Shantou University, Shantou, Guangdong 515063, China

^2^State Key Laboratory of Optoelectronic Materials and Technologies, Sun Yat-sen University, Guangzhou 510275, China

^3^ School of Physics and Optoelectronic Engineering, Foshan University, Foshan 528000, China

^*^Corresponding authors: [sysuhhx@163.com](mailto:sysuhhx@163.com), [stszjy@mail.sysu.edu.cn](mailto:stszjy@mail.sysu.edu.cn)

Rigorous derivation of the Point spread function


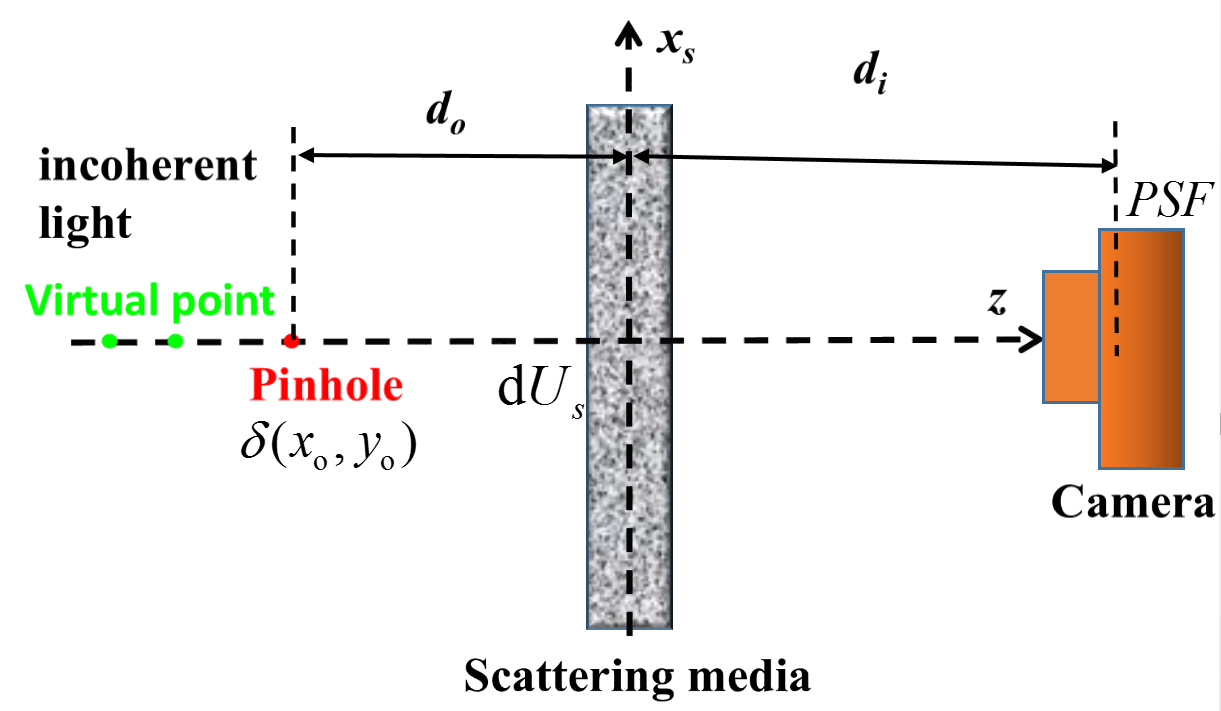


**Figure S1.** Schematic of theoretical analysis of the PSF.

Here we present a rigorous derivation of PSFs from different objective plane. As shown in Fig. S1. A pinhole (red point) is located on the object plane and illuminated by a spatial incoherent light source. After its transmission light passing through a thin scattering medium, a speckle pattern is formed on the camera plane. The light field before the scattering media is written in the form of a sphere wave,

$\begin{matrix} dU_{s}(x_{s},y_{s};x_{o0},y_{o0})=\frac{e^{ikd_{o}}}{i\lambda d_{o}}\int\int_{-\infty}^{\infty} \delta(x_{o},y_{o})e^{\frac{ik}{2d_{o}}[(x_{s}-x_{o})^{2}+(y_{s}-y_{o})^{2}]}dx_{o}dy_{o} \\ =\frac{e^{ikd_{o}}}{i\lambda d_{o}}e^{\frac{ik}{2d_{o}}[x_{s}^{2}+y_{s}^{2}]} \end{matrix}$. (S1)

When treating the thin scattering media as a random phase mask *TM*(*x_s_*, *y_s_*) ^1^, the light field distribution on the imaging plane is^2, 3^,

$\begin{matrix} h(x_{i},y_{i};x_{o},y_{o})\text{=}-\frac{e^{ik(d_{o}+d_{i})}}{\lambda^{2}d_{o}d_{i}}e^{\frac{ik}{2d_{i}}(x_{i}^{2}+y_{i}^{2})}\cdot\int\int_{-\infty}^{\infty} \text{TM}(x_{s},y_{s})e^{\frac{ik}{2f}(x_{s}^{2}+y_{s}^{2})}e^{-ik\left[\frac{x_{i}}{d_{i}}x_{s}+\frac{y_{i}}{d_{i}}y_{s}\right]}dx_{s}dy_{s} \\ \text{=C}\cdot F\text{\{TM}(x_{s},y_{s})e^{\frac{ik}{2f}(x_{s}^{2}+y_{s}^{2})}\text{\}}_{x_{s}\to\frac{x_{\text{i}}}{\lambda d_{i}},y_{s}\to\frac{y_{\text{i}}}{\lambda d_{i}}} \end{matrix}$, (S2)

where *d_i_* and *d_o_* are image distance and object distance respectively, *f* is the focal length of the “scattering lens” which meets the formula of object-image distance (1/*f*=1/*d_i_*+1/*d_o_*). $F\text{\{\}}$is a symbol of Fourier transformation.

The intensity of PSF under incoherent illumination is recorded, which can be written as the square of the module of the light field^4^,

$\begin{matrix} PSF(x_{i},y_{i}\text{;}x_{\text{o}},y_{\text{o}})=h_{I}(x_{i},y_{i})=\left|h(x_{i},y_{i}\text{;}x_{\text{o}},y_{\text{o}})\right|^{2} \\ \text{=}\left|CF\left\{\text{TM}(x_{s},y_{s})e^{\frac{ik}{2f}(x_{s}^{2}+y_{s}^{2})}\right\}_{x_{s}\to\frac{x_{\text{i}}}{\lambda d_{i}},y_{s}\to\frac{y_{\text{i}}}{\lambda d_{i}}}\right|^{2} \\ \text{=}C^{2}F\left\{R\left\{\left[\text{TM}(x_{s},y_{s})e^{\frac{ik}{2f}(x_{s}^{2}+y_{s}^{2})}\right]\right\}\right\}_{x_{s}\to\frac{x_{\text{i}}}{\lambda d_{i}},y_{s}\to\frac{y_{\text{i}}}{\lambda d_{i}}} \\ =C^{2}F\left\{e^{-\frac{ik}{2f}(x_{s}^{2}+y_{s}^{2})}F_{a}^{-1}\left[\text{TM}(\alpha,\beta)\cdot\text{TM}^{*}(\alpha-x_{s},\beta-y_{s})\right]_{\alpha\to\frac{x_{s}}{2\lambda f}\text{,}\beta\to\frac{y_{s}}{2\lambda f}}\right\}_{x_{s}\to\frac{x_{\text{i}}}{\lambda d_{i}},y_{s}\to\frac{y_{\text{i}}}{\lambda d_{i}}} \end{matrix}$, (S3)

where $R\text{\{\}}$ is a symbol of autocorrelation function and the autocorrelation theorem$F\text{\{}R[f(x,y)]\text{\}=|}F\text{\{}f(x,y)\text{\}|}^{2}$ is applied for the deduction.

The quadratic phase term in equation (S3) can be rewritten in the form of Fourier transform,

$\begin{matrix} e^{-\frac{ik}{2f}(x_{s}^{2}+y_{s}^{2})}=F_{a}^{-1}\text{\{}F_{a}[e^{-\frac{ik}{2f}(x_{s}^{2}+y_{s}^{2})}]_{\frac{x_{s}}{2\lambda f}\to\alpha,\frac{y_{s}}{2\lambda f}\to\beta}\}_{\alpha\to\frac{x_{s}}{2\lambda f}\text{,}\beta\to\frac{y_{s}}{2\lambda f}} \\ =F_{a}^{-1}\text{[}-i\sqrt{1/4\lambda f}e^{-\frac{i}{4\lambda f}(\alpha^{2}+\beta^{2})}]_{\alpha\to\frac{x_{s}}{2\lambda f}\text{,}\beta\to\frac{y_{s}}{2\lambda f}} \end{matrix}$. (S4)

The PSF becomes,

, (S5)

wher$A\left(\alpha,\beta\right)=\sqrt{1/4i\lambda f}e^{-\frac{ik}{8f}\left(\alpha^{2}+\beta^{2}\right)}*\left[TM\left(\alpha,\beta\right)\cdot TM\left(\alpha-x_{s},\beta-y_{s}\right)\right]$. When the object distance translated to *dʹ_o_*, the new PSF becomes,

$PSF'(x_{i},y_{i})=F\text{\{}F_{a}^{-1}\text{[A(}\alpha,\beta\text{)}]_{\alpha\to\frac{x_{s}}{2\lambda f'}\text{,}\beta\to\frac{y_{s}}{2\lambda f'}}\}_{x_{s}\to\frac{x_{\text{i}}}{\lambda d_{i}},y_{s}\to\frac{y_{\text{i}}}{\lambda d_{i}}}$, (S6)

where the transform variables in $F_{a}^{-1}$ is charging to $\alpha\to\frac{x_{1}}{2\lambda f'}$ and $\beta\to\frac{y_{1}}{2\lambda f'}$(1/*fʹ*=1/*d_i_*+1/*d*ʹ*_o_*).

The new PSF is equal to the original one except the scaling in transform variable,

$PSF'(x_{i},y_{i})=m^{2}PSF(mx_{i},my_{i})$, (S7)

where the scaling factor $m=f/f'$ $=\frac{(d_{i}+d_{o})d_{o}^{'}}{(d_{i}+d_{o}^{'})d_{o}}$ *.*

The difference between PSFs in various object planes is only on the size scale. Once a PSF is obtained, virtual PSFs of other object planes can be calculated by resizing the PSF with a scaling factor *m*.

**A brief derivation on the axial FOV with the improved speckle rescaling method**

In this section we derived the axial FOV of a scattering-lens based deconvolution (correlation) imaging. As defined by Katz et al^5^, the axial FOV, Δ*z_FOV_*, is the maximum axial shift of a point source for which the generated speckle pattern on the other side of the medium is still correlated with the one generated by the original point-source position (inside the “memory effect” range). In Katz’s model, a spatial light modulator (SLM) can perfectly compensate the phase aberration of the light field derived from the reference point source passing through the scattering medium. The SLM and the scattering medium were combined as a correlating lens.

As it is shown in Fig. S2, a point source at the left hand side of the scattering medium changes along the axis from distance of *d_o_* to *d_o_*-Δ*d_o_*. It leads to an addition of quadratic phase to the wave incident on the medium surface:

, (S8)

where $k_{o}=2\pi/\lambda$ . The gradient of this quadratic phase envelope is:

. (S9)

**Figure S2.** Geometry used for estimating the axial field of view $\Delta z_{FOV}$.

By connecting the maximal phase gradient to the maximal angle of transverse FOV, the axial FOV can be obtained as^5^,

$\Delta z_{FOV}=2\left(\Delta d_{o}\right)_{\max}=2\frac{d_{o}}{D}FOV$. (S10)

This result is also valid for the setup in Fig. S2 if the scattering lens maintains the same focal length and the zooming effect on the speckle correlation is omitted. The axial magnification of a scattering lens, when referring to a thin lens with fix focal length, is$\alpha\text{=}\frac{\Delta d_{i}}{\Delta d_{o}}\text{=}\frac{d_{i}^{2}}{d_{o}^{2}}$. A translation of point source along the axial by Δ*d_o_* will leads to a translation of its image by$\Delta d_{i}\text{=}\frac{d_{i}^{2}}{d_{o}^{2}}\Delta d_{o}$. The angle of the incident light changes by$\Delta\theta_{o}=\rho\Delta d_{o}/d_{o}^{2}$. It will leads to the transmission light tilts by an angel of Δ*θ_i_*. According to Memory Effect [Ref. 21-22 of the content], Δ*θ_i_=*Δ*θ_o_*. The speckle intensity correlation for the objects on plane *d_o_* and plane *d_o_*-Δ*d_o_* can be calculated by

$C\left(\Delta\theta_{o},L\right)=\left[\frac{k_{o}\Delta\theta_{o}L}{\sinh\left(k_{o}\Delta\theta_{o}L\right)}\right]^{2}$, (S11)

where *L* is an effective thickness. The maximum tilted angle is approximately$\left(\Delta\theta_{o}\right)_{\max}\sim\lambda/2\pi L$, and corresponded to$FOV\sim\lambda d_{o}/\pi L$.

While in our model, the camera is placed on plane *d_i_* only. The zooming effect can be cancelled out by rescaling the speckle, but speckles degradation between plane *d_i_* and plane *d_i_*+Δ*d_i_* should be considered. A strict derivation can be referred to Van Cittert-Zernike theorem. Without loss of generality, only points on axial are considered. As for a same scattering medium, the field angle of axial points on plane *d_i_* and plane *d_i_*+Δ*d_i_* change by Δ*θ_i_*. So the speckle intensity correlation between plane *d_i_* and plane *d_i_*+Δ*d_i_* can be estimated by equation (S11).

Finally, the correlation of speckles generated by point sources on *d_o_* and *d_o_*-Δ*d_o_* for our rescaling method is proportional to$\left[k_{o}\Delta\theta_{o}L/\sinh\left(k_{o}\Delta\theta_{o}L\right)\right]^{4}$. From this conclusion, we obtain the maximal axial translation:

$\Delta z_{FOV}=1.4d_{o}\frac{FOV}{D}$. (S12)

In our experiment, *d_o_*=15cm, *D*=5mm. FOV is tested by another experiment, result turns out to be 1.5mm. So axial FOV is approximately 61mm, which is larger than the improved DOF 36.6 mm in experiment.

**Reference：**

1. Schott, S., Bertolotti, J., Léger, J. F., Bourdieu, L., & Gigan, S., Characterization of the angular memory effect of scattered light in biological tissues, *Optics Express* **23**(10), 13505-3516 (2015).

2. Freund I., Looking through walls and around corners, *Physica A: Statistical Mechanics and its Applications* **168**(1), 49-65 (1990).

3. Freund, I., Rosenbluh M., & Feng, S., Memory Effects in Propagation of Optical Waves through Disordered Media, *Physical Review Letters* **61**(20), 2328-2331 (1988).

4. Zhuang, H., He, H,. Xie, X., & Zhou, J., High speed color imaging through scattering media with a large field of view, *Scientific Reports* **6**, 32696 (2016).

5. Katz, O., Small, E. & Silberberg, Y. Looking around corners and through thin turbid layers in real time with scattered incoherent light. *Nature Photon* **6**, 549-553 (2012).
